# An up-converting phosphor technology-based lateral flow assay for rapid detection of major mycotoxins in feed: Comparison with enzyme-linked immunosorbent assay and high-performance liquid chromatography-tandem mass spectrometry

**DOI:** 10.1371/journal.pone.0250250

**Published:** 2021-04-16

**Authors:** Fenghua Zhu, Beibei Zhang, Lianqin Zhu

**Affiliations:** 1 College of Animal Science and Technology, Qingdao Agricultural University, Qingdao, People’s Republic of China; 2 College of Veterinary Medicine, Qingdao Agricultural University, Qingdao, People’s Republic of China; Tallinn University of Technology, ESTONIA

## Abstract

Current methods for detection of mycotoxin in feed are time-consuming and tedious. An up-converting phosphor technology-based lateral flow (UPT-LF) assay system is a new emerging technique for analytes detection. The aim of this study was to compare the performance of UPT-LF, an enzyme-linked immunosorbent assay (ELISA) and high-performance liquid chromatography-tandem mass spectrometry (HPLC-MS/MS) for detecting aflatoxin B_1_ (AFB_1_), zearalenone (ZEN) and deoxynivalenol (DON) in feed. The results showed that the use of UPT-LF for AFB_1_, ZEN and DON detection exhibited the following: limits of detection of 3, 50 and 200 μg/kg; average recoveries of 104.39%, 102.94% and 103.65%; and precision of 13.96%, 13.71% and 12.56%; respectively. UPT-LF required 45 min to determine one mycotoxin and 1.5 h to determine three mycotoxins in a sample, which took the shortest time. Besides, there were positive correlations between the UPT-LF, ELISA and HPLC/MS/MS methods. In conclusion, UPT-LF can be used to detect and quantify AFB_1_, ZEN and DON in feed samples. Though the sensitivity, accuracy and precision of UPT-LF are inferior to those of HPLC-MS/MS and ELISA, the UPT-LF assay is the most convenient and rapid technique for on-site detection among the three methods.

## Introduction

Mycotoxins are secondary metabolites produced by filamentous fungi, especially those belonging to the genera *Aspergillus*, *Penicillium*, *Alternaria*, and *Fusarium*. Mycotoxins pose a high risk to the health of livestock and humans due to their toxigenic, carcinogenic, immunogenic, neurogenic, and mutagenic properties [1]. Currently, approximately 300 to 400 mycotoxins have been identified [2]. Among these mycotoxins, aflatoxin, zearalenone (ZEN), and deoxynivalenol (DON) are considered the most important due to serious safety problems and their prevalence in feeds [2, 3]. Aflatoxins are produced by *Aspergillus* species of fungi, and the four most abundant forms in contaminated feedstuffs are aflatoxin B_1_ (AFB_1_), B_2_, G_1_ and G_2_. Of these, AFB_1_ is the most common and most carcinogenic toxin. Even low concentrations of AFB_1_ intake by humans or livestock will cause severe acute or chronic liver injury, including hepatic necrosis, cirrhosis, and carcinoma [4]. DON and ZEN are toxic secondary metabolites mainly produced by *Fusarium* species. ZEN is an estrogen analog that can bind to estrogen receptors, causing severe damage to the reproductive system and having carcinogenic potential in humans and animals [2, 5, 6]. DON has more severe effects on monogastric animals and may cause nausea, emesis, anorexia, diarrhea and gastrointestinal hemorrhaging, as well as increase pathogen susceptibility and decrease performance [7–9].

Considering the toxicity of mycotoxins, many countries have set strict standards regarding the maximum levels of these mycotoxins in feed. The limits vary with the type of mycotoxin, animal species, raw materials, feed, intended use and country. For example, the European Commission set the maximum levels of AFB_1_ for all feed materials and complete feedstuffs in a range of 5 to 20 μg/kg [10]. ZEN should be no more than 100 μg/kg in cereals and 350 μg/kg in corn [11]. The maximum limits for DON have been regulated at 900 μg/kg in complete feedstuffs for pigs [12]. In China, the maximum limits for AFB_1_ range from 5 to 50 μg/kg in feed, and the maximum guidance values for ZEN in corn and complete feed is 500 μg/kg [13]. Additionally, the maximum limits for DON range from 1000 to 5000 μg/kg in complete feed [14].

Due to strict rules and regulations, more rapid, accurate, sensitive, simple and inexpensive analytical techniques for mycotoxins are required by feed-producing companies. High-performance liquid chromatography-tandem mass spectrometry (HPLC-MS/MS) and enzyme-linked immunosorbent assay (ELISA) techniques have been used to detect and quantify mycotoxins in feed. HPLC-MS/MS is sensitive and produces reliable results and has been accepted as an official method. However, this method requires expensive instrumentation and a complex sample pretreatment and is time-consuming, which is unsuitable for rapid on-site detection. With the benefits of simplicity, adaptability, sensitivity, and selectivity, ELISA has been developed as a popular technique [15]. However, this method involves multiple incubations and washing steps, which is also time-consuming. Moreover, inappropriate operation may cause false positive or false negative results.

In the last two decades, an up-converting phosphor technology-based lateral flow (UPT-LF) assay has emerged as a new technique for analyte detection. Immunochromatographic assay, also called lateral flow immunoassay, has attracted considerable attention due to the advantages of being a low-cost and simple-to-perform technique that provides a rapid and sensitive detection of various analytes. Up-converting phosphor (UCP) is a kind of lanthanide-containing, submicrometer-sized ceramic particle that possesses a unique optical property of up-converting infrared excitation light to emit visible light [16]. UPT reporters are 10- to 100-fold more sensitive than assays using colloidal gold or colored latex beads [17]. Combining UCP with a lateral flow immunoassay exhibits little background interference from complex matrices, which ensures its high sensitivity and stability. To date, the UPT-LF assay has been widely used for the sensitive detection of drugs of abuse [18], nucleic acids [19], interferon-γ [20], and various infectious pathogens and foodborne pathogens, such as *Escherichia coli* [18], *Coxiella burnetii* [21] and *Yersinia pestis* [22].

Several studies have been reported regarding the application of UPT-LF for rapid quantitative detection of AFB_1_ in crop samples [23]. However, few research has been conducted to compare the performance of UPT-LF, HPLC-MS/MS and ELISA for mycotoxin detection. Thus, we describe an UPT-LF assay for rapid detection of AFB_1_, ZEN and DON in feed and comprehensively compare its performance with that of HPLC-MS/MS and ELISA.

## Material and methods

### Instruments and reagents

A mechanical shaker was purchased from Shanghai Shiping Experimental Equipment Co., Ltd. (SPH-21B, Shanghai, China). A ten-thousandth electronic balance was provided by Ohaus International Trading (Shanghai) Co., Ltd. (AR2140, Shanghai, China). A microplate reader was purchased from ThermoFisher Scientific Inc. (MK3, Waltham, MA). HPLC-MS/MS was performed on a 1290 Infinity ultra-high-performance liquid chromatograph (UHPLC) system (Agilent Technologies Inc., Santa Clara, CA, USA) coupled to a 6460 triple quadrupole mass spectrometer (QQQ) equipped with a Jet Stream ion source (Agilent Technologies Inc., Santa Clara, CA, USA). The UPT-based biosensor was obtained from Beijing Hotgen Biotech Co., Ltd. (UPT-3A, Beijing, China).

ELISA kits for AFB_1_, ZEN and DON quantification were provided by Romer Labs (MO, USA). UCP immunoassay kits for AFB_1_, ZEN and DON quantification were provided by Beijing Hotgen Biotech Co., Ltd. (Beijing, China). Standard solutions of AFB_1_, ZEN and DON were purchased from Pribolab Biological Technical Company (Qingdao, China). Water was purified using a Milli-Q system (Millipore Corporation, Bedford, MA). All other chemicals and solvents were of HPLC grade.

### Sampling

Feed samples were collected according to the sampling method of GB/T 14669.1–2005 [24]. The feed ingredient samples including corn, soybean meal, peanut meal, wheat bran, distiller’s dried grain with solubles (DDGS), corn protein powder, cottonseed meal, rapeseed meal, corn germ meal, alfalfa, wheat, fish meal and corn straw and mixed feed samples including pig concentrated feed, laying hen concentrated feed, broiler complete feed, cow complete feed, pig complete feed, meat duck complete feed and laying hen complete feed were randomly collected from feed-producing companies and animal farms across the Shandong Province of China. Additionally, 5 different samples were collected per feed. The sources of these feed samples are shown in S1 Table. Overall, 100 samples were collected for mycotoxin detection. Furthermore, corn without AFB_1_, ZEN and DON detectable by HPLC-MS/MS was considered mycotoxin-free corn and was used as a blank sample for method validation. Mycotoxin-free corn and 100 feed samples were ground using a laboratory crusher and passed through a 0.85 mm sieve before being stored at -20°C for further analysis.

### Determination by UPT-LF

**Fig 1:**
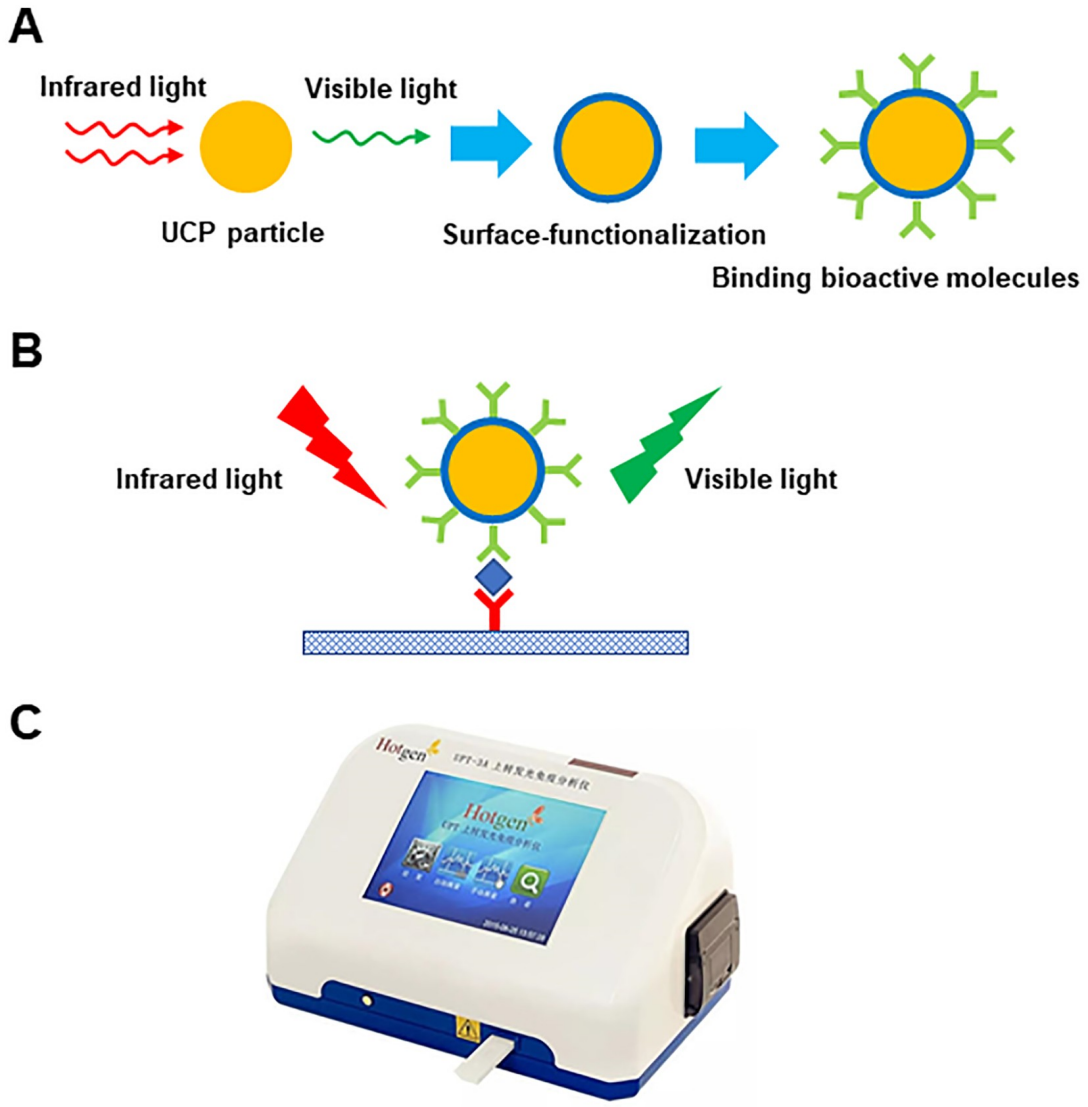
Schematic diagram of UPT-LF test card for the detection of feed sample. (A) UCP particle was functionalized and conjugated with anti-mycotoxin antibody. (B) UCP-mycotoxin complex bound to the capturing antibody on the test strip. (C) The test card was scanned by UPT-3A biosensor.

Fig 1 shows the schematic diagram of UPT-LF method for the detection of feed sample. UCP is a kind of lanthanide-containing, submicrometer-sized ceramic particle that can absorb infrared light and emit visible light. After covered with a thin and uniform layer of SiO_2_ and surface-modified with derivatives of silanylation reagents, UCP particle was conjugated with anti-mycotoxin antibody. The analytical membranes of UPT-LF strips were functionalized with another anti-mycotoxin antibody. The more mycotoxin in the sample, the more fluorescence conjugate binds to the capturing antibodies on the test strip that leads to an increase in fluorescence intensity. The detailed principle and operational procedure of the UPT-based biosensor have been described previously [25]. Briefly, 3 g (accurate to 0.0001 g) of ground feed was placed into a flask, and 15 mL of methanol-water (70:30, v/v; for AFB_1_ and ZEN extraction) or distilled water (for DON extraction) was added to the flask. Next, the solution was shaken for 5 min on a mechanical shaker. After standing for 5 min, the solution was filtered through filter paper (no. 1, Whatman, Clifton, NJ), and 200 μL of filtrate was diluted with 600 μL of sample diluent from the UCP immunoassay kits. Then, the sample solution was considered ready for the UPT-LF assay on the UPT-based biosensor. UCP particles are used in the UPT immunoassay as the biolabel. The temperature should be within a range of 20°C ~25°C, and the humidity should be below 50% before the instrument is turned on. Fifteen minutes after the instrument was turned on, a parameter card was inserted, and a calibration was conducted according to the sample types. Next, a test card was placed horizontally, and 100 μL of sample solution was added to the sample well of the test card. After standing for 15 min, the test card was scanned for the bar code at the bottom to obtain the interface information. Then, the test card was inserted into the UPT-based biosensor for mycotoxin detection.

### Determination by ELISA

Briefly, 20 g (accurate to 0.0001 g) of ground feed was placed in a 250-mL flask with 100 mL of methanol-water (70:30, v/v). The flask was then shaken for 3 min using a mechanical shaker. After standing for 5 min, the solution was filtered through filter paper (no. 1, Whatman, Clifton, NJ), and 10 mL of the filtrate was diluted with 50 mL of methanol-water (70:30, v/v). Mycotoxin detection was carried out according to the manufacturer’s instructions for ELISA kits. The results were calculated using Romer Log/Logit software provided by the manufacturer.

### Determination by HPLC-MS/MS

In this experiment, a 5 g (accurate to 0.0001 g) ground sample was placed into a flask, and 25 mL of acetonitrile-water (86:14, v/v) was added to the flask. Next, the solution was shaken for 1 h using a mechanical shaker, filtered with quantitative filter paper and then filtered with a 0.22-μm-pore-size membrane filter (Jinlong Co., Ltd., Tianjin, China). The filtrate was placed into a sterile centrifuge tube and analyzed using the HPLC-MS/MS system. For the HPLC-MS/MS procedure, separation was carried out in a C18 column (2.1 mm×50 mm, 1.8 μm particle size; Agilent Technologies Inc., USA), and the column temperature was 33°C. For AFB_1_ and DON, chromatographic elution was performed with a mobile phase consisting of methyl alcohol (part A) and 0.2% formic acid-water in ultrapure water (v/v, part B). The gradient elution at a 0.2 mL/min flow rate started at 80% B and was decreased to 50% B after 2 min and 0% B after 6 min via a linear gradient mode. Next, 100% A at a 0.3 mL/min flow rate was held for 2 min, and then 80% B at a 0.2 mL/min flow rate was started at 8.01 min and held for 2 min. For ZEN, chromatographic elution was performed with a mobile phase consisting of methyl alcohol (part A) and 1 mmol/L ammonium acetate in ultrapure water (part B). An isocratic elution at a 0.2 mL/min flow rate started at 20% B and was held for 1.4 min. Next, 100% A at a 0.3 mL/min flow rate was started at 1.41 min and held for 2 min. Then, 20% B at a 0.2 mL/min flow rate was started at 3.42 min and held for 2 min. The injection volume was 2 μL. The mass spectrometer was operated using a turbo ion-spray ionization source configured for electrospray ionization (ESI) in positive and negative switching ion modes, and acquisition was conducted using multiple reaction monitoring with a dwell time of 100 ms. The ion voltages for the positive and negative ion modes were both 4,500 V. Resolutions at Q1 and Q3 were set to units. Argon gas (99% purity) was used in the ESI source and the collision cell. The mass parameters of the ESI detection mode, precursor ion, product ion, cone voltage, fragmentor voltage, cell accelerator voltage and collision energy for each mycotoxin are summarized in Table 1.

**Table 1:** HPLC-MS/MS parameters for the determination of multiple mycotoxins.

Mycotoxin^a^	ESI detection mode	Precursor ion (m/z)	Product ion (m/z)	Cone voltage (V)	Fragmentor voltage (V)	Cell accelerator voltage (V)	Collision energy (eV)
AFB_1_	+	313.1	241.0	40	180	3	40
ZEN	-	317.1	175.1	15	190	3	15
DON	+	297.1	249.1	24	80	3	8

^*a*^AFB_1_, aflatoxin B_1_; ZEN, zearalenone; DON, deoxynivalenol.

### Method validation

#### Sensitivity

The limits of detection (LODs) of UPT-LF and HPLC-MS/MS were estimated according to the China National Standard (GB/T 5009.1–2003) [26]. Mycotoxin-free corn powder was used as a blank sample and was spiked at five different mycotoxin concentrations, with three replicates per concentration. The calibration curves were constructed by a linear regression analysis. The LOD of UPT-LF was calculated using the formula LOD_U_ = Ks/b, where K is a coefficient determined according to a certain degree of confidence and number of replicates, which can be obtained by checking the t-distribution quantile table in the China National Standard (GB/T 4889–2008) [27]; s is the standard deviation of the blank samples; and b is the slope of the calibration curve measured by UPT-LF. The LOD of HPLC-MS/MS was calculated as LOD_H_ = 3N/b, where N is the instrumental noise level and b is the slope of the calibration curve measured by HPLC-MS/MS. The LOD of ELISA was calculated as LOD_E_ = 0.01/b, where b is the slope of the calibration curve measured by ELISA.

#### Recovery studies

To assess accuracy, recovery tests were carried out by spiking mycotoxin-free corn powder with each mycotoxin standard solution at different concentrations. For AFB_1_ detection, the standard solution of AFB_1_ was spiked into a corn powder at final concentrations of 20, 30, 40, 60 and 80 μg/kg. For ZEN detection, the standard solution of ZEN was spiked into a corn powder at final concentrations of 100, 200, 300, 400 and 800 μg/kg. For DON detection, the standard solution of DON was spiked into a corn powder at final concentrations of 200, 400, 800, 1600 and 3200 μg/kg. Corn powder was used as a blank sample. Each sample was divided into 15 replicates, processed separately and subsequently analyzed. The procedures were carried out according to the UPT-LF, ELISA and HPLC/MS/MS methods described above. The recovery rate was expressed as the deviation of experimental from nominal concentration values in percent, recovery (%) = (measured value–blank value)/theorical value×100.

#### Precision

The precision was determined by spiking mycotoxin-free corn powder with each mycotoxin standard solution at medium concentrations (60 μg/kg AFB_1_, 400 μg/kg ZEN and 800 μg/kg DON). Each sample with 15 replicates was then tested to obtain precision. The precision was expressed as the relative standard deviation (RSD), RSD (%) = (standard deviation/mean)×100.

### Real sample determination

Approximately 200 g of air-dried sample per feed was retained using a quarter method, ground and passed through a 0.85 mm sieve, put into zipper bags and then stored at -20°C. The determinations of AFB_1_, ZEN and DON were conducted according to the UPT-LF, ELISA and HPLC-MS/MS methods described above. The mean, median and maximum mycotoxin concentrations in the feed samples detected by the three methods are listed in Tables. The median is a more representative parameter than the mean and standard deviation when the distribution of data presents a positive skew. Additionally, to show the most serious mycotoxin contamination, we listed the maximum value. These two parameters are beneficial for the statistical description of the data.

### Statistical analysis

SPSS statistical software (version 22.0, IBM Corp., Chicago, IL, USA) was used for statistical analysis. Differences in recovery rates and mycotoxin concentrations in feed measured by the UPT-LF, ELISA and HPLC-MS/MS methods were analyzed by one-way ANOVA with a post hoc Duncan’s multiple comparisons test. When one method failed to detect the mycotoxin concentration in feed samples, Student’s *t*-test was used to compare the mean concentration obtained from the other two methods. Pearson’s correlation analysis was used to analyze the correlations between the mycotoxin concentrations of feed samples measured by the three methods. The recovery rate and mycotoxin concentration results are presented as the mean ± standard deviation. Differences were considered significant at *P* < 0.05.

## Results and discussion

### Sensitivity, accuracy and precision

The sensitivity (Table 2) was evaluated by calculating LODs. The LODs of the three mycotoxins by HPLC-MS/MS (0.001 μg/kg AFB_1_, 0.01 μg/kg ZEN, and 1 μg/kg DON) were much lower than those of the other two methods (3 μg/kg AFB_1_, 50 μg/kg ZEN, 200 μg/kg DON by UPT-LF and 2 μg/kg AFB_1_, 25 μg/kg ZEN, and 250 μg/kg DON by ELISA). The LODs of each mycotoxin by the ELISA method were similar to those of the UPT-LF method. The China National Standard (GB13078-2017) [28] states that the minimum limits of AFB_1_, ZEN and DON in feed are 10 μg/kg, 100 μg/kg, and 1000 μg/kg, respectively. The LODs of AFB_1_, ZEN and DON by the UPT-LF method were 3 μg/kg, 50 μg/kg, and 200 μg/kg, respectively, which were satisfactory for the detection requirements of the three mycotoxins in feed. Zhao et al. [23] reported that the LOD of AFB_1_ in corn by UPT-LF was 3 μg/kg, which was similar to our results. The recovery and precision tests were performed by spiking corn powder samples with standard solutions (Table 2). For UPT-LF, the recovery rates and precision of AFB_1_, ZEN and DON were determined: the recovery rates of AFB_1_ ranged from 81.71% to 127.23%, with a mean value of 104.39%; the recovery rates of ZEN ranged from 76.47% to 129.24%, with a mean value of 102.94%; and the recovery rates of DON ranged from 79.06% to 122.50%, with a mean value of 103.65%. Furthermore, the RSD values of AFB_1_, ZEN, and DON were 13.96%, 13.71% and 12.56%, respectively. For ELISA, the recovery rates and precision of AFB_1_, ZEN and DON were also determined: the recovery rates were 101.75±8.99%, 99.23±8.69%, and 100.43±7.82%, respectively. Additionally, the RSD values of AFB_1_, ZEN, and DON were revealed to be 8.89%, 8.93% and 8.02%, respectively. For HPLC-MS/MS, the recovery rates and precision of AFB_1_, ZEN and DON were also assessed: the recovery rates were 100.88±2.67%, 101.46±2.72% and 100.22±2.34%, respectively. Moreover, the associated RSD values of AFB_1_, ZEN, and DON were demonstrated to be 2.67%, 1.89% and 2.80%, respectively. The recovery rates and precision of the HPLC-MS/MS method were better than those of the other two methods, and the recovery rates and precision of ELISA were better than those of UPT-LF. Similarly, Beyene et al. [29] reported that HPLC had higher precision, selectivity, and sensitivity for the detection of AFB_1_ in feed samples than those of ELISA. However, there was no significant difference in mean recovery among the three methods (*P* < 0.05). All the results met the requirements of recovery rate and precision by the National Criterion of China (GB/T 23182–2008) [27], in which the average recovery rate was required to range from 50% to 120% and RSD was required to be less than 20%. The above data demonstrated that all the analytical methods were reliable, accurate and reproducible for the identification and quantification of AFB_1_, ZEN and DON.

**Table 2:** Sensitivity, accuracy and precision of UPT-LF, ELISA and HPLC-MS/MS methods for detection of multiple mycotoxins.

Mycotoxin^a^	Method	LOD^b^ (μg/kg)	Mean recovery (%)	Minimum recovery (%)	Maximum recovery (%)	RSD^c^ (%)
AFB_1_	UPT-LF	3	104.39±14.21	81.71	127.23	13.96
	ELISA	2	101.75±8.99	87.94	124.64	8.89
	HPLC-MS/MS	0.001	100.88±2.67	92.50	104.66	2.67
ZEN	UPT-LF	50	102.94±15.40	76.47	129.24	13.71
	ELISA	25	99.23±8.69	83.51	116.93	8.93
	HPLC-MS/MS	0.01	101.46±2.72	96.59	105.02	1.89
DON	UPT-LF	200	103.65±12.65	79.06	122.50	12.56
	ELISA	250	100.43±7.82	89.00	115.55	8.02
	HPLC-MS/MS	1	100.22±2.34	94.85	104.97	2.80

Means with different superscripts are significantly different within a column (*P* < 0.05). Data are expressed as Mean ± SD (n = 15).

^*a*^AFB_1_, aflatoxin B_1_; ZEN, zearalenone; DON, deoxynivalenol.

^*b*^LOD, limit of detection.

^*c*^RSD, relative standard deviation.

### Detection time

The detection times of the three methods are shown in Table 3. From sample treatment to data analysis, the UPT-LF method took the shortest period time (0.75 h) to detect one mycotoxin in a sample, followed by the HPLC-MS/MS method (2 h), and finally the ELISA method, which took the longest period of time (3 h). When detecting all three mycotoxins in a sample, the UPT-LF method still took the shortest period of time (1.5 h), followed by the HPLC method (3.5 h) and the ELISA method (5 h). To prevent or minimise mycotoxicosis in animals, rapid, simple, sensitive and accurate methods for mycotoxin detection in feed are still needed. HPLC-MS/MS and ELISA are time consuming and not readily available for on-site detection [23, 30]. Compared with the HPLC and ELISA methods, the UPT-LF method is quicker and easier to operate. Rapid detection is one of the obvious advantages of UPT-LF method which has been reported in numerous researches [21, 31, 32].

**Table 3:** Detection time of UPT-LF, ELISA and HPLC-MS/MS methods for detection of mycotoxins.

Mycotoxin number	Method	Time (h)
One	UPT-LF	0.75
	ELISA	3
	HPLC-MS/MS	2
Three	UPT-LF	1.5
	ELISA	5
	HPLC-MS/MS	3.5

### Comparison of three methods for AFB_1_ detection in feed ingredients

Table 4 illustrates the AFB_1_ concentrations in feed ingredients measured by UPT-LF, ELISA and HPLC-MS/MS. The positive rates of AFB_1_ in various feed ingredients were 100% as measured by the three methods, except for the results of soybean meal and cottonseed meal. The contamination of AFB_1_ in soybean meal was undetectable by UPT-LF. The maximum concentrations of AFB_1_ in the soybean meal measured by ELISA and HPLC-MS/MS were 2.74 and 1.85 μg/kg, respectively. However, the China National Standard (GB13078-2017) [28] states that the limit of AFB_1_ in soybean meal is 30 μg/kg. The results indicated that all of the AFB_1_ concentrations measured by the three methods did not exceed the China National Standard, and UPT-LF was acceptable for the purpose of rapid screening of AFB_1_-contaminated soybean meal. There were no significant differences in the average levels of AFB_1_ in peanut meal among the UPT-LF, ELISA and HPLC-MS/MS methods, along with the results of corn protein powder, rapeseed meal and fish meal (*P* > 0.05). The mean levels of AFB_1_ in corn, cottonseed meal, corn germ meal, alfalfa and corn straw by the UPT-LF method were significantly higher than those of the ELISA and HPLC-MS/MS methods (*P* < 0.05). However, no significant difference in AFB_1_ concentration was observed between the ELISA and HPLC-MS/MS methods in the above feed samples (*P* > 0.05). This result indicated that there was a centain difference between UPT-LF and the other two methods in the accuracy of AFB_1_ detection in the above feed ingredients.

**Table 4:** Aflatoxin B_1_ contamination in feed ingredients measured by UPT-LF, ELISA and HPLC-MS/MS methods.

Feed ingredient	Method	Positive rate (%)	Mean value (μg/kg)	Median value (μg/kg)	Maximum value (μg/kg)
Corn	UPT-LF	100	3.60±0.45^a^	3.52	4.31
	ELISA	100	1.72±0.43^b^	1.45	2.27
	HPLC-MS/MS	100	1.26±0.47^b^	1.08	2.10
Soybean meal	UPT-LF	—	—	—	—
	ELISA	100	2.25±0.41^a^	2.18	2.74
	HPLC-MS/MS	100	1.36±0.35^b^	1.19	1.85
Peanut meal	UPT-LF	100	80.79±15.45	85.39	94.81
	ELISA	100	66.04±14.91	65.21	81.36
	HPLC-MS/MS	100	56.53±33.91	54.41	100.71
Wheat bran	UPT-LF	100	14.56±2.84^a^	15.38	17.08
	ELISA	100	8.22±1.85^b^	8.70	10.07
	HPLC-MS/MS	100	5.00±14.90^c^	4.96	7.55
DDGS^a^	UPT-LF	100	27.69±5.74^a^	26.91	31.18
	ELISA	100	24.42±4.65^ab^	24.15	31.08
	HPLC-MS/MS	100	20.90±1.90^b^	21.04	22.95
Corn protein powder	UPT-LF	100	25.89±8.61	27.33	35.16
	ELISA	100	16.64±28.33	3.85	67.30
	HPLC-MS/MS	100	11.32±9.11	8.69	26.30
Cottonseed meal	UPT-LF	100	86.98±10.53^a^	84.25	98.56
	ELISA	80	59.55±23.01^b^	56.88	84.61
	HPLC-MS/MS	100	45.94±4.34^b^	45.21	51.09
Rapeseed meal	UPT-LF	100	8.34±2.28	7.96	10.96
	ELISA	100	6.37±1.97	6.09	8.38
	HPLC-MS/MS	100	6.86±2.13	7.79	8.21
Corn germ meal	UPT-LF	100	161.66±15.66^a^	154.36	186.34
	ELISA	100	137.60±3.62^b^	137.41	143.20
	HPLC-MS/MS	100	136.73±5.18^b^	138.18	143.12
Alfalfa	UPT-LF	100	53.37±7.90^a^	54.28	62.35
	ELISA	100	45.34±4.75^b^	46.39	49.96
	HPLC-MS/MS	100	49.21±11.36^b^	47.23	61.87
Wheat	UPT-LF	100	40.38±13.97^a^	33.11	63.79
	ELISA	100	24.53±4.36^b^	23.71	30.71
	HPLC-MS/MS	100	35.24±8.64^ab^	33.60	46.04
Fish meal	UPT-LF	100	25.27±5.25	24.52	32.93
	ELISA	100	21.72±5.80	22.15	28.70
	HPLC-MS/MS	100	24.53±9.26	26.89	33.45
Corn straw	UPT-LF	100	38.37±12.82^a^	33.58	160.12
	ELISA	100	21.42±3.04^b^	20.81	26.36
	HPLC-MS/MS	100	21.56±7.53^b^	19.02	34.37

Means with different superscripts are significantly different within a column (*P* < 0.05). Data are expressed as Mean ± SD (n = 5).

^*a*^DDGS, distiller’s dried grain with solubles (corn).

### Comparison of three methods for ZEN detection in feed ingredients

As shown in Table 5, 100% of the feed ingredient samples were contaminated by ZEN when measured by the UPT-LF, ELISA and HPLC-MS/MS methods, except that the ZEN contamination was undetectable in cottonseed meal and rapeseed meal by the UPT-LF and ELISA methods. The maximum concentrations of ZEN in the cottonseed meal and rapeseed meal measured by HPLC-MS/MS were 27.37 and 23.97 μg/kg, respectively. According to the China National Standard (GB13078-2017) [28], the maximum permitted concentration of ZEN in cottonseed meal and rapeseed meal is 1000 μg/kg. The results indicated that UPT-LF and ELISA could satisfy the requirements of screening ZEN-contaminated cottonseed meal and rapeseed meal. No significant difference was identified in the average levels of ZEN in the corn protein power, alfalfa, fish meal and corn straw among the three methods (*P* > 0.05). The average levels of ZEN in the corn, soybean meal, peanut meal, wheat bran, DDGS, and wheat by the UPT-LF method were significantly higher than those of the HPLC-MS/MS method (*P* < 0.05), and the results of corn germ meal measured by UPT-LF were significantly lower than those of the HPLC-MS/MS method (*P* < 0.05). Additionally, there was no significant difference in the average levels of ZEN in peanut meal, DDGS and corn germ meal when comparing UPT-LF with ELISA (*P* > 0.05).

**Table 5:** Zearalenone contamination in feed ingredients measured by UPT-LF, ELISA and HPLC-MS/MS methods.

Feed ingredient	Method	Positive rate (%)	Mean value (μg/kg)	Median value (μg/kg)	Maximum value (μg/kg)
Corn	UPT-LF	100	36.30±5.80^b^	35.62	42.52
	ELISA	100	53.48±5.43^a^	53.45	60.84
	HPLC-MS/MS	100	24.69±2.74^c^	23.60	28.42
Soybean meal	UPT-LF	100	127.00±18.49^a^	119.57	147.70
	ELISA	100	60.68±8.13^b^	57.09	70.75
	HPLC-MS/MS	100	64.12±8.57^b^	63.39	74.23
Peanut meal	UPT-LF	100	38.53±15.29^a^	41.08	52.30
	ELISA	100	33.57±7.56^ab^	31.22	45.20
	HPLC-MS/MS	100	22.37±0.62^b^	22.58	22.97
Wheat bran	UPT-LF	100	73.30±10.05^a^	76.12	85.31
	ELISA	100	50.03±23.12^b^	44.07	89.26
	HPLC-MS/MS	100	27.19±6.55^c^	24.53	38.86
DDGS^a^	UPT-LF	100	36.61±10.19^a^	33.51	51.20
	ELISA	100	29.27±5.43^ab^	26.92	35.17
	HPLC-MS/MS	100	24.44±1.22^b^	24.45	25.53
Corn protein powder	UPT-LF	100	138.41±131.57	75.99	370.98
	ELISA	100	229.92±295.86	84.92	752.17
	HPLC-MS/MS	100	59.37±20.70	39.08	149.54
Cottonseed meal	UPT-LF	—	—	—	—
	ELISA	—	—	—	—
	HPLC-MS/MS	100	24.31±1.88	23.80	27.37
Rapeseed meal	UPT-LF	—	—	—	—
	ELISA	—	—	—	—
	HPLC-MS/MS	100	23.58±0.50	23.83	23.97
Corn germ meal	UPT-LF	100	291.27±56.61^b^	302.84	350.91
	ELISA	100	307.60±52.33^b^	327.77	373.07
	HPLC-MS/MS	100	394.54±46.97^a^	381.74	468.85
Alfalfa	UPT-LF	100	226.76±32.20	215.57	279.26
	ELISA	100	255.79±65.90	258.78	340.63
	HPLC-MS/MS	100	285.39±102.05	266.33	434.99
Wheat	UPT-LF	100	202.60±49.19^a^	207.42	266.35
	ELISA	100	89.46±51.44^b^	75.09	169.01
	HPLC-MS/MS	100	96.05±51.91^b^	102.43	168.77
Fish meal	UPT-LF	100	54.29±4.84	53.87	60.73
	ELISA	100	57.06±37.00	46.79	118.43
	HPLC-MS/MS	100	61.80±34.55	47.26	121.46
Corn straw	UPT-LF	100	161.40±96.48	203.06	248.56
	ELISA	100	135.00±42.05	152.07	171.31
	HPLC-MS/MS	100	139.65±42.04	154.23	179.96

Means with different superscripts are significantly different within a column (*P* < 0.05). Data are expressed as Mean ± SD (n = 5).

^*a*^DDGS, distiller’s dried grain with solubles (corn).

### Comparison of three methods for DON detection in feed ingredients

The DON levels in feed ingredients measured by the three methods are presented in Table 6. The positive rates of DON in the feed ingredients were 100% as measured by UPT-LF, ELISA and HPLC-MS/MS methods, except that the DON contamination was undetectable in peanut meal by the UPT-LF method. The maximum concentrations of DON in the peanut meal measured by ELISA and HPLC-MS/MS were 214.51 and 200.31 μg/kg, respectively. The China maximum concentration of DON in peanut meal is 5000 μg/kg [28]. The data demonstrated that UPT-LF could satisfy the requirement of screening DON-contaminated peanut meal. No significant difference was identified in the average levels of DON in the corn, soybean meal, wheat bran, DDGS, corn protein power, rapeseed meal, corn germ meal and wheat among the three methods (*P* > 0.05). Besides, Median values of DON in the above feed ingredients among the three methods were highly similar, so were the maximum values. This suggested that UPT-LF assay showed good accuracy for detection of DON in these feed ingredients. There was no significant difference in the average levels of DON in cottonseed meal, alfalfa and fish meal between UPT-LF and HPLC-MS/MS (*P* < 0.05). The average levels of DON in the corn straw by the UPT-LF and ELISA methods were significantly higher than those of the HPLC-MS/MS method (*P* < 0.05). There was no significant difference in the average level of DON in corn straw between the UPT-LF and ELISA methods (*P* > 0.05). The average levels of DON in cottonseed meal and fish meal by the UPT-LF method were significantly higher than those of the ELISA method (*P* < 0.05), and the average levels of DON in alfalfa by the UPT-LF method were significantly lower than those of the ELISA method (*P* < 0.05).

**Table 6:** Deoxynivalenol contamination in feed ingredients measured by UPT-LF, ELISA and HPLC-MS/MS methods.

Feed ingredient	Method	Positive rate (%)	Mean value (μg/kg)	Median value (μg/kg)	Maximum value (μg/kg)
Corn	UPT-LF	100	276.00±95.29	210.00	390.00
	ELISA	100	267.47±112.60	230.64	400.38
	HPLC-MS/MS	100	266.36±106.58	210.63	396.61
Soybean meal	UPT-LF	100	344.00±24.27	380.00	400.00
	ELISA	100	318.41±43.49	301.26	374.22
	HPLC-MS/MS	100	232.54±68.72	368.41	376.84
Peanut meal	UPT-LF	—	—	—	—
	ELISA	100	174.79±32.22	180.23	214.51
	HPLC-MS/MS	100	185.22±13.08	186.31	200.31
Wheat bran	UPT-LF	100	302.00±219.02	270.00	530.00
	ELISA	100	310.15±213.21	251.30	534.86
	HPLC-MS/MS	100	261.28±178.14	261.28	509.34
DDGS^a^	UPT-LF	100	224.00±26.08	210.00	270.00
	ELISA	100	171.71±71.94	162.34	261.20
	HPLC-MS/MS	100	240.89±43.73	246.37	299.84
Corn protein powder	UPT-LF	100	398.00±69.43	360.00	510.00
	ELISA	100	298.46±131.01	286.97	425.33
	HPLC-MS/MS	100	418.02±170.75	415.38	681.52
Cottonseed meal	UPT-LF	100	214.00±11.40^a^	210.00	230.00
	ELISA	100	151.70±20.28^b^	150.34	171.23
	HPLC-MS/MS	100	180.12±37.72^ab^	180.34	240.13
Rapeseed meal	UPT-LF	100	214.00±145.02	210.00	230.00
	ELISA	100	194.89±13.43	192.46	215.20
	HPLC-MS/MS	100	195.83±15.22	192.33	220.34
Corn germ meal	UPT-LF	100	2758.00±474.84	2860.00	3250.00
	ELISA	100	2857.39±332.67	2965.41	3142.05
	HPLC-MS/MS	100	2144.77±1185.18	2683.41	3064.81
Alfalfa	UPT-LF	100	1063.00±207.29^b^	1090.00	1340.00
	ELISA	100	1949.31±311.74^a^	1823.64	2341.35
	HPLC-MS/MS	100	964.24±50.66^b^	956.32	1023.35
Wheat	UPT-LF	100	1128.00±169.91	1090.00	1380.00
	ELISA	100	1016.45±237.84	1024.30	1350.36
	HPLC-MS/MS	100	1081.65±43.70	1071.21	1142.05
Fish meal	UPT-LF	100	742.00±144.81^a^	680.00	980.00
	ELISA	100	465.53±182.31^b^	440.58	698.13
	HPLC-MS/MS	100	632.06±30.99^ab^	641.20	667.59
Corn straw	UPT-LF	100	286.00±55.50^a^	260.00	380.00
	ELISA	100	289.68±76.25^a^	266.38	410.25
	HPLC-MS/MS	100	264.37±30.27^b^	254.61	317.05

Means with different superscripts are significantly different within a column (*P* < 0.05). Data are expressed as Mean ± SD (n = 5).

^*a*^DDGS, distiller’s dried grain with solubles (corn).

### Comparison of three methods for AFB_1_ detection in feed mixtures

Table 7 shows the AFB_1_ levels in the feed mixture measured by the UPT-LF, ELISA and HPLC-MS/MS methods. AFB_1_ was detected in 100% of the feed mixture samples measured by the UPT-LF, ELISA and HPLC-MS/MS methods, except that 60% of the meat duck complete feed was AFB_1_-contaminated, as measured by the UPT-LF method. No significant difference was identified in the average levels of AFB_1_ in the pig and laying hen concentrated feeds and the broiler, cow, pig and meat duck complete feeds among the three methods (*P* > 0.05), which suggested that these three methods had good consistency in detecting AFB_1_ concentration of the above feeds. The average level of AFB_1_ in laying hen complete feed by the UPT-LF method was significantly higher than those of the ELISA and HPLC-MS/MS methods (*P* < 0.05), and no significance was obtained in the results measured by the ELISA and HPLC-MS/MS methods (*P* > 0.05).

**Table 7:** Aflatoxin B_1_ contamination in feed mixtures measured by UPT-LF, ELISA and HPLC-MS/MS methods.

Feed mixture	Method	Positive rate (%)	Mean value (μg/kg)	Median value (μg/kg)	Maximum value (μg/kg)
Pig concentrated feed	UPT-LF	100	7.33±0.19	7.33	7.54
	ELISA	100	9.43±2.87	11.05	11.95
	HPLC-MS/MS	100	9.16±2.80	10.62	11.48
Laying hen concentrated feed	UPT-LF	100	35.84±13.50	29.66	54.17
	ELISA	100	24.27±9.43	22.73	35.73
	HPLC-MS/MS	100	23.00±10.78	19.30	37.12
Broiler complete feed	UPT-LF	100	19.65±6.38	22.01	24.87
	ELISA	100	22.36±9.48	20.34	34.33
	HPLC-MS/MS	100	17.68±8.02	15.52	30.61
Cow complete feed	UPT-LF	100	36.51±10.16	33.69	51.88
	ELISA	100	26.07±7.67	24.88	38.95
	HPLC-MS/MS	100	26.61±8.00	23.03	39.51
Pig complete feed	UPT-LF	100	7.26±1.77	6.95	10.27
	ELISA	100	6.64±1.69	6.38	9.53
	HPLC-MS/MS	100	5.86±1.52	5.71	8.22
Meat duck complete feed	UPT-LF	60	10.59±12.26	22.43	25.09
	ELISA	100	15.07±3.39	11.02	27.34
	HPLC-MS/MS	100	14.92±7.88	11.19	28.56
Laying hen complete feed	UPT-LF	100	30.22±3.72^a^	29.57	34.94
	ELISA	100	17.79±4.18^b^	17.59	21.73
	HPLC-MS/MS	100	17.85±4.02^b^	17.75	21.64

Means with different superscripts are significantly different within a column (*P* < 0.05). Data are expressed as Mean ± SD (n = 5).

### Comparison of three methods for ZEN detection in feed mixtures

As shown in Table 8, ZEN contamination was found in all feed mixture samples measured by the UPT-LF, ELISA and HPLC-MS/MS methods, except that 60% of pig complete feed samples were contaminated by ZEN when measured by the UPT-LF method. This meant that the AFB_1_ concentrations of 2 samples from the pig complete feed were undetectable by the UPT-LF method. However, the maximum concentrations of ZEN in the pig complete feed measured by ELISA and HPLC-MS/MS were 99.16 and 34.83 μg/kg, respectively. The limits for ZEN in pig complete feed regulated by the China National Standard (GB13078-2017) are 250 μg/kg [28]. Our results showed that UPT-LF could be used to detect ZEN in pig complete feed for screening purposes. No significant difference was identified in the average levels of ZEN in the pig and laying hen concentrated feeds or the broiler and cow complete feeds among the three methods (*P* > 0.05). The average level of ZEN in pig complete feed measured by the ELISA method was significantly higher than that of the HPLC-MS/MS method (*P* < 0.05), but there was no difference in the result obtained by the UPT-LF method when compared with those of ELISA or HPLC-MS/MS (*P* > 0.05). The average levels of ZEN in meat duck and laying hen complete feeds by the UPT-LF method were significantly higher than those of the ELISA and HPLC-MS/MS methods (*P* < 0.05), and no significance was observed in the results detected by the ELISA and HPLC-MS/MS methods (*P* < 0.05).

**Table 8:** Zearalenone contamination in feed mixtures measured by UPT-LF, ELISA and HPLC-MS/MS methods.

Feed mixture	Method	Positive rate (%)	Mean value (μg/kg)	Median value (μg/kg)	Maximum value (μg/kg)
Pig concentrated feed	UPT-LF	100	73.63±9.98	68.83	88.97
	ELISA	100	73.35±34.10	87.92	109.40
	HPLC-MS/MS	100	84.96±21.97	79.49	110.98
Laying hen concentrated feed	UPT-LF	100	325.65±61.38	333.27	418.52
	ELISA	100	224.05±109.74	246.84	338.82
	HPLC-MS/MS	100	238.05±122.33	253.80	394.23
Broiler complete feed	UPT-LF	100	76.75±29.83	63.45	128.85
	ELISA	100	92.96±27.15	91.60	135.33
	HPLC-MS/MS	100	78.51±31.99	99.63	103.94
Cow complete feed	UPT-LF	100	81.89±20.97	73.79	119.02
	ELISA	100	88.05±34.49	98.62	126.23
	HPLC-MS/MS	100	103.97±27.66	88.82	150.17
Pig complete feed	UPT-LF	60	41.56±42.18^ab^	56.06	99.31
	ELISA	100	71.12±16.77^a^	67.62	99.16
	HPLC-MS/MS	100	29.04±3.35^b^	27.94	34.83
Meat duck complete feed	UPT-LF	100	103.14±19.09^a^	93.94	127.65
	ELISA	100	34.50±21.84^b^	23.50	69.57
	HPLC-MS/MS	100	52.91±10.70^b^	57.40	61.03
Laying hen complete feed	UPT-LF	100	70.95±18.90^a^	66.53	96.65
	ELISA	100	34.92±9.17^b^	29.85	49.07
	HPLC-MS/MS	100	43.33±3.25^b^	42.96	48.19

Means with different superscripts are significantly different within a column (*P* < 0.05). Data are expressed as Mean ± SD (n = 5).

### Comparison of three methods for DON detection in feed mixtures

As shown in Table 9, the positive rates of DON in the feed mixtures were 100% as measured by the UPT-LF, ELISA and HPLC-MS/MS methods, with the exception of the results in pig concentrated feed by UPT-LF and laying hen complete feed by UPT-LF and ELISA. DON was detectable in 80% of the pig concentrated feed samples measured by UPT-LF; it was undetectable in 1 sample. Additionally, the UPT-LF and ELISA methods failed to detect any DON contamination in all the laying hen complete feed. The maximum DON concentrations in pig concentrated feed by ELISA and HPLC-MS/MS were 680.35 and 681.23 μg/kg, respectively. The maximum DON concentration in laying hen complete feed by HPLC-MS/MS was 24.91 μg/kg. The limit for DON in pig concentrated feed and laying hen complete feed is 3000 μg/kg [28]. The results indicated that UPT-LF was acceptable for detecting DON in pig concentrated feed and laying hen complete feed for screening purposes. No significant difference was identified in the average levels of DON in the pig and laying hen concentrated feeds or the broiler, cow, and meat duck complete feeds. among the three methods (*P* > 0.05), which indicated that the results of UPT-LF were consistent with the other two methods for the DON concentration in the above feeds. The average level of DON in pig complete feed by ELISA was significantly lower than those of the UPT-LF and HPLC-MS/MS methods (*P* < 0.05), and no significance was observed in the results between the UPT-LF and HPLC-MS/MS methods (*P* > 0.05).

**Table 9:** Deoxynivalenol contamination in feed mixtures measured by UPT-LF, ELISA and HPLC-MS/MS methods.

Feed mixture	Method	Positive rate (%)	Mean value (μg/kg)	Median value (μg/kg)	Maximum value (μg/kg)
Pig concentrated feed	UPT-LF	80	482.50±246.52	520.00	680.00
	ELISA	100	384.14±241.40	374.20	680.35
	HPLC-MS/MS	100	517.90±254.20	581.23	681.23
Laying hen concentrated feed	UPT-LF	100	424.00±174.73	450.00	680.00
	ELISA	100	398.01±130.45	453.28	512.69
	HPLC-MS/MS	100	445.24±192.66	462.53	642.30
Broiler complete feed	UPT-LF	100	302.00±132.93	210.00	480.00
	ELISA	100	221.95±134.60	150.12	421.37
	HPLC-MS/MS	100	345.27±125.51	271.03	521.94
Cow complete feed	UPT-LF	100	858.00±427.22	970.00	1230.00
	ELISA	100	825.11±461.80	990.34	1231.72
	HPLC-MS/MS	100	806.09±446.44	980.34	1233.67
Pig complete feed	UPT-LF	100	276.00±67.31^a^	260.00	380.00
	ELISA	100	209.14±41.89^b^	180.34	256.31
	HPLC-MS/MS	100	304.86±50.30^a^	331.28	356.91
Meat duck complete feed	UPT-LF	100	204.00±145.02	200.00	410.00
	ELISA	100	210.82±136.39	174.20	450.36
	HPLC-MS/MS	100	202.24±115.80	145.31	403.69
Laying hen complete feed	UPT-LF	—	—	—	—
	ELISA	—	—	—	—
	HPLC-MS/MS	100	17.81±4.42	16.34	24.91

Means with different superscripts are significantly different within a column (*P* < 0.05). Data are expressed as Mean ± SD (n = 5).

### Correlation between three methods

The correlations among the mycotoxin results determined by UPT-LF, ELISA and HPLC-MS/MS are shown in Table 10. There were significant positive linear correlations among the values of mycotoxins measured by UPT-LF, HPLC-MS/MS and ELISA (r > 0.68, *P* < 0.01). The ELISA and HPLC-MS/MS results were highly correlated for the detection of AFB_1_ (r = 0.828), ZEN (r = 0.814) and DON (r = 0.828), which indicated a close agreement between the results of ELISA and HPLC for the analysis of mycotoxin contamination in feeds. Similarly, Beyene et al. [29] reported a positive correlation coefficient of 0.84 between HPLC and ELISA in the AFB_1_ concentrations of feed samples. UPT-LF and HPLC-MS/MS showed good correlations for concentrations of AFB_1_ (r = 0.855), ZEN (r = 0.776) and DON (r = 0.834) in feeds. There were positive correlations for the detection of AFB_1_ (r = 0.793), ZEN (r = 0.684) and DON (r = 0.886) between the UPT-LF and ELISA methods. The above data indicated that UPT-LF could be used to detect and quantify AFB_1_, ZEN and DON in feed samples.

**Table 10:** Correlations between the UPT-LF, ELISA and HPLC-MS/MS methods.

Mycotoxin^a^	Method	Correlation coefficient (r)^b^		
		UPT-LF	ELISA	HPLC-MS/MS
AFB_1_	UPT-LF	1	0.793**	0.855**
	ELISA	0.793**	1	0.828**
	HPLC-MS/MS	0.855**	0.828**	1
ZEN	UPT-LF	1	0.684**	0.776**
	ELISA	0.684**	1	0.814**
	HPLC-MS/MS	0.776**	0.814**	1
DON	UPT-LF	1	0.886**	0834**
	ELISA	0.886**	1	0.828**
	HPLC-MS/MS	0834**	0.828**	1

^*a*^AFB_1_, aflatoxin B_1_; ZEN, zearalenone; DON, deoxynivalenol.

^b^**P < 0.01, Pearson’s correlation analysis.

## Conclusion

UPT-LF can be used to detect and quantify AFB_1_, ZEN and DON in feed samples. Among the three methods, HPLC-MS/MS exhibits the highest sensitivity, recovery and precision, followed by ELISA. However, HPLC-MS/MS is expensive, time-consuming and requires trained, skilled technicians, while ELISA involves tedious procedures and contains matrix interference. The UPT-LF method may be the most convenient and quickest technique for on-site detection among the three methods, which is more suitable for rapid screening purposes.

## Supporting information

S1 Table Sources of the 100 feed samples. CO: corn; SM: soybean meal; PM: peanut meal; WB: wheat bran; DDGS: distiller’s dried grain with solubles; CPP: corn protein powder; CM: cottonseed meal; RM: rapeseed meal; CGM: corn germ meal; AL: alfalfa; WH: wheat; FM: fish meal; CS: corn straw; PCTF: pig concentrated feed; LHCTF: laying hen concentrated feed; BCF: broiler complete feed; CCF: cow complete feed; PCF: pig complete feed; MDCF: meat duck complete feed; LHCF: laying hen complete feed.
(DOCX)

## Abbreviations

AFB_1_: aflatoxin B_1_
DDGS: distiller’s dried grain with solubles
DON: deoxynivalenol
ELISA: enzyme-linked immunosorbent assay
ESI: electrospray ionization
HPLC-MS/MS: high-performance liquid chromatography-tandem mass spectrometry
LOD: limit of detection
QQQ: triple quadrupole mass spectrometer
RSD: relative standard deviation
UCP: up-converting phosphor
UHPLC: ultra-high-performance liquid chromatograph
UPT-LF: up-converting phosphor technology-based lateral flow
ZEN: zearalenone
