# Supplementary material for: An up-converting phosphor technology-based lateral flow assay for rapid detection of major mycotoxins in feed: Comparison with enzyme-linked immunosorbent assay and high-performance liquid chromatography-tandem mass spectrometry
Source: PLoS One. 2021 Apr 16;16(4):e0250250. doi: 10.1371/journal.pone.0250250 (PMC8051755; doi:10.1371/journal.pone.0250250)
Supplement: S1 Table — CO: corn; SM: soybean meal; PM: peanut meal; WB: wheat bran; DDGS: distiller’s dried grain with solubles; CPP: corn protein powder; CM: cottonseed meal; RM: rapeseed meal; CGM: corn germ meal; AL: alfalfa; WH: wheat; FM: fish meal; CS: corn straw; PCTF: pig concentrated feed; LHCTF: laying hen concentrated feed; BCF: broiler complete feed; CCF: cow complete feed; PCF: pig complete feed; MDCF: meat duck complete feed; LHCF: laying hen complete feed. (DOCX) [file pone.0250250.s001.docx]

**S1 Table. Sources of the 100 feed samples.**

| Sampling location | CO | SM | PM | WB | DDGS | CPP | CM | RM | CGM | AL | WH | FM | CS | PCTF | LHCTF | BCF | CCF | PCF | MDCF | LHCF | Total |
| --- | --- | --- | --- | --- | --- | --- | --- | --- | --- | --- | --- | --- | --- | --- | --- | --- | --- | --- | --- | --- | --- |
| Qingdao | 1 | 1 | 1 | 1 |  |  |  |  |  |  |  |  |  | 1 | 1 | 1 | 1 |  |  |  | 8 |
| Jinan |  |  | 1 | 1 | 1 | 1 |  |  |  |  |  |  |  |  |  | 1 | 1 | 1 | 1 |  | 8 |
| Yantai |  |  |  |  | 1 | 1 | 1 | 1 |  |  |  |  |  |  |  |  | 1 | 1 | 1 | 1 | 8 |
| Weifang | 1 |  |  |  |  |  | 1 | 1 | 1 | 1 |  |  | 1 |  | 1 |  |  |  |  | 1 | 8 |
| Linyi |  | 1 | 1 | 1 |  | 1 |  |  |  |  | 1 | 1 |  | 1 | 1 |  |  |  |  |  | 8 |
| Jining | 1 |  |  |  |  |  |  |  |  |  |  | 1 |  |  |  | 1 |  |  | 1 | 1 | 5 |
| Zibo |  |  |  |  | 1 |  |  |  |  |  | 1 | 1 |  | 1 |  | 1 |  |  |  |  | 5 |
| Weihai |  |  | 1 | 1 |  |  | 1 |  | 1 |  |  |  | 1 |  |  |  |  |  |  |  | 5 |
| Dongying |  | 1 |  |  | 1 |  |  |  |  |  |  | 1 | 1 |  | 1 |  |  |  |  |  | 5 |
| Rizhao | 1 |  |  |  |  |  |  | 1 |  | 1 |  |  |  |  |  |  | 1 |  |  | 1 | 5 |
| Taian |  |  | 1 |  |  | 1 |  |  |  |  | 1 |  |  | 1 |  |  |  | 1 |  |  | 5 |
| Binzhou |  |  |  | 1 | 1 |  | 1 |  |  |  |  |  | 1 |  |  |  |  |  | 1 |  | 5 |
| Zaozhuang | 1 | 1 |  |  |  |  |  | 1 | 1 |  |  |  |  |  | 1 |  |  |  |  |  | 5 |
| Dezhou |  |  |  |  |  |  | 1 |  |  | 1 |  | 1 |  |  |  |  | 1 | 1 |  |  | 5 |
| Liaocheng |  | 1 |  |  |  | 1 |  |  | 1 |  | 1 |  |  |  |  | 1 |  |  |  |  | 5 |
| Laiwu |  |  |  |  |  |  |  | 1 |  | 1 | 1 |  |  |  |  |  |  | 1 | 1 |  | 5 |
| Heze |  |  |  |  |  |  |  |  | 1 | 1 |  |  | 1 | 1 |  |  |  |  |  | 1 | 5 |
| Total | 5 | 5 | 5 | 5 | 5 | 5 | 5 | 5 | 5 | 5 | 5 | 5 | 5 | 5 | 5 | 5 | 5 | 5 | 5 | 5 | 100 |

CO: corn; SM: soybean meal; PM: peanut meal; WB: wheat bran; DDGS: distiller’s dried grain with solubles; CPP: corn protein powder; CM: cottonseed meal; RM: rapeseed meal; CGM: corn germ meal; AL: alfalfa; WH: wheat; FM: fish meal; CS: corn straw; PCTF: pig concentrated feed; LHCTF: laying hen concentrated feed; BCF: broiler complete feed; CCF: cow complete feed; PCF: pig complete feed; MDCF: meat duck complete feed; LHCF: laying hen complete feed.
